# Long-Haul COVID Patients: Prevalence of POTS Are Reduced but Cerebral Blood Flow Abnormalities Remain Abnormal with Longer Disease Duration

**DOI:** 10.3390/healthcare10102105

**Published:** 2022-10-21

**Authors:** C. (Linda) M. C. van Campen, Frans C. Visser

**Affiliations:** Stichting Cardiozorg, Planetenweg 5, 2132 HN Hoofddorp, The Netherlands

**Keywords:** orthostatic intolerance, long-haul COVID, tilt testing, cerebral blood flow, postural orthostatic tachycardia (POTS), extra cranial Doppler echography

## Abstract

Background: Postural orthostatic tachycardia syndrome (POTS) has been described early after the onset of the COVID-19 infection, but also orthostatic hypotension (OH). In the present study, we hypothesized that orthostatic intolerance decreases over time. Methods: In 29 long-haul COVID-19 (LHC) patients, a tilt test was performed, including measurements of cerebral blood flow (CBF) by extracranial Doppler. The time interval between the onset of infection and the tilt test varied between 3 and 28 months. Results: In the first 12 months after the infection, 71% of the LHC patients showed POTS and after 24 months none of them. In the first 12 months, 29% of patients had a normal heart rate and blood pressure response (normHRBP) and after 24 months 75% (distribution of POTS, OH, and a normHRBP over time: *p* < 0.0001). Linear regression showed that, over time, there was a decrease in the abnormal CBF during the tilt (*p* = 0.024) but remained abnormal. Conclusion: In LHC patients, hemodynamic abnormalities of a tilt test change over time. Patients studied early after the onset of the disease mainly exhibit POTS, but patients studied later in the time course mainly show a normHRBP or OH. In addition, the abnormal CBF reduction improves over time, but CBF remains abnormal.

## 1. Introduction

Since the outbreak of the pandemic by the particularly contagious respiratory virus SARS-CoV-2 (COVID-19) virus in March 2020, the early onset of orthostatic intolerance with postural orthostatic tachycardia syndrome (POTS) has been noted and described in several case reports [1,2,3,4,5,6,7,8,9,10,11,12,13]. POTS was found in the very early days of the COVID-19 infection, even on Day 1 of the infection [10], and can persist for more than two years [13]. However, also other forms of orthostatic intolerance, such as orthostatic hypotension (OH) and syncope [1,10], have been described. Moreover, recovery of POTS and OH after the onset of the disease have been described [1]. In our initial case series of 10 long-haul COVID-19 patients, all showed POTS during tilt testing, while later patients could show orthostatic hypotension or a normal heart rate and blood pressure (normHRBP) response during tilt testing. In addition, patients spontaneously reported that their heart rates decreased over time. Therefore, we hypothesized that the type of orthostatic intolerance abnormality (POTS, OH, or syncope) during a tilt test may change over time after the onset of the disease.

Therefore, the aim of our study was to correlate long-haul COVID-19 disease duration with the prevalence of various types of hemodynamic abnormalities during a tilt test in 29 patients. Furthermore, we demonstrated in the initial 10 long-haul COVID-19 patients with POTS an abnormal cerebral blood flow reduction during the upright phase of the tilt test compared to cerebral blood flow in the supine position [13]. Thus, the second aim of the study was to explore whether the long-haul COVID-19 disease duration correlated with cerebral blood flow reduction, hypothesizing that cerebral blood flow reduction may improve over time.

## 2. Materials and Methods

### 2.1. Participants

In the period from December 2020 to March 2022, 29 patients with long-haul COVID complaints were seen at our outpatient clinic being referred for the evaluation of orthostatic intolerance and/or dysautonomia. In the first 11 patients, there was a clinical suspicion of a SARS-CoV-2 infection; in the remaining patients, the diagnosis was confirmed by a serological test. In the beginning of 2020 (from February to June 2020), serological tests were not performed or discouraged due to poor availability. These patients were included in the study if the clinical diagnosis was made by a pulmonologist, internist, general practitioner, or the general health service of the cities. All patients underwent a tilt table test with cerebral blood flow measurement for quantification of their orthostatic intolerance. In none of the long-haul COVID patients was there any other explanation for the symptoms found. During the tilt test study, patients were not taking medication that could affect heart rate and/or blood pressure. Finally, prior to the tilt table examination, orthostatic intolerance complaints in daily life were assessed [13,14]. On the basis of questions about complaints such as dizziness, lightheadedness, previous syncope, nausea, sweating, etc., and also on the basis of questions about triggers for the development of these complaints, such as standing in a line, showering, etc., the diagnosis of orthostatic intolerance in daily life was made. None of the patients used heart rate or blood-lowering medication, as well as SSRIs and painkillers. Although many patients used supplements, we did not ask for the type of supplements and therefore could not address the influence of supplements. The study was conducted in accordance with the Helsinki Declaration. All patients gave written permission for the use of their data. The use of clinical information was approved by the medical ethics committee of the Slotervaart Hospital in Amsterdam (P1736).

### 2.2. Tilt Test Protocol to Quantify Orthostatic Intolerance

The methodology used for the tilt table test was previously described [15,16]. For details, see Appendix A.

### 2.3. Extracranial Doppler Measurements for Determination of Cerebral Blood Flow

Measurements were carried out as described earlier [15,16]. For more details see Appendix A.

### 2.4. Statistics

Data were analyzed using the GraphPad Prism version 6.05 statistics program (GraphPad Software, La Jolla, CA, USA). All continuous data were tested for normal distribution with the D’Agostino and Pearson omnibus normality test and were presented as mean and standard deviation (SD) or median with interquartile range (IQR) where appropriate. Nominal data were compared with the Chi-square test. Between-group comparison was performed by the unpaired *t*-test or Mann–Whitney U test where appropriate. A linear regression analysis was performed on the relationship between disease duration and cerebral blood flow reduction during the tilt test. A value of *p* < 0.05 was considered significant.

## 3. Results

The study population consisted of 29 long-haul COVID-19 patients. Mean age was 39 (12) years. In 13 POTS, in 5 OH, and in 11 patients, a normal heart rate and blood pressure response were observed. The median time interval between the onset of COVID-19 symptoms and the tilt test was 18 months with a range between 3 and 28 months. Table 1 shows the clinical characteristics of these three long-haul COVID-19 patients. Clinical characteristics were not different, including the presence of orthostatic intolerance symptoms in daily life.

Table 2 shows the hemodynamic results of the tilt table test. The heart rate at the end of the tilt test was significantly higher in patients with POTS compared to patients with a normal heart rate and blood pressure (ANOVA post-hoc Tukey’s test: *p* = 0.006). Other measurements were not significantly different.

**Figure 1:**
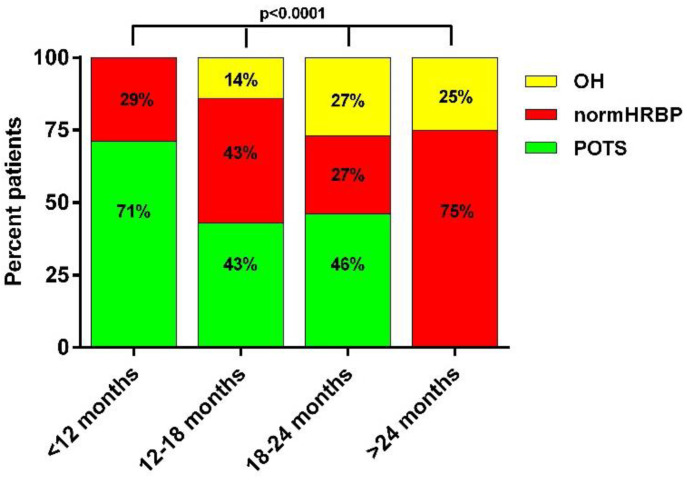
Frequencies of hemodynamic tilt test results in long-haul COVID-19 patients: relation with disease duration. OH: orthostatic hypotension; normHRBP: normal heart rate and blood pressure response; POTS: postural orthostatic tachycardia syndrome.

Figure 1 shows the percentage of POTS, OH, and patients with a normal heart rate and blood pressure in four different time intervals after the onset of symptoms of COVID-19, the intervals being less than 12 months, between 12 and 18 months, between 18 and 24 months, and larger than 24 months. The frequency of POTS among the three hemodynamic profiles declined over time, with 71% of patients showing POTS in the first 12 months and none after 24 months. In contrast, the prevalence of OH and of a normal heart rate and blood pressure increased over time, with the highest prevalence of a normal heart rate and blood pressure response (75%) after 24 months. These changes in distribution patterns over time were significantly different (ANOVA: *p* < 0.0001).

**Figure 2:**
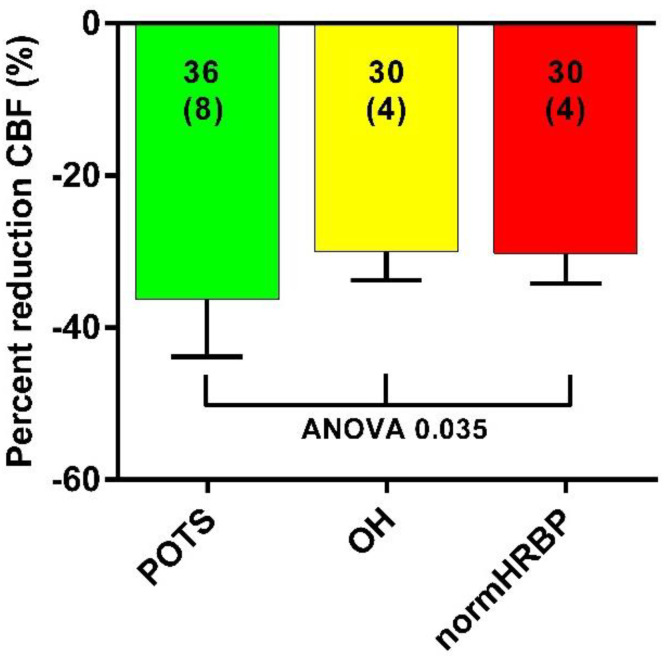
Cerebral blood flow reduction in long-haul COVID-19 patients with POTS, with orthostatic hypotension, and with a normal heart rate and blood pressure during the tilt test. CBF: cerebral blood flow; OH: orthostatic hypotension; normHRBP: normal heart rate and blood pressure response during the tilt test.

Figure 2 shows the percentage reduction in cerebral blood flow in the three groups. There was a significant difference in the percent cerebral blood flow reduction in patients with POTS compared to the two other groups: 36 (8) versus 30 (4) in patients with OH, and versus 30 (4) in patients with a normal heart rate and blood pressure response (ANOVA: *p* = 0.035).

**Figure 3:**
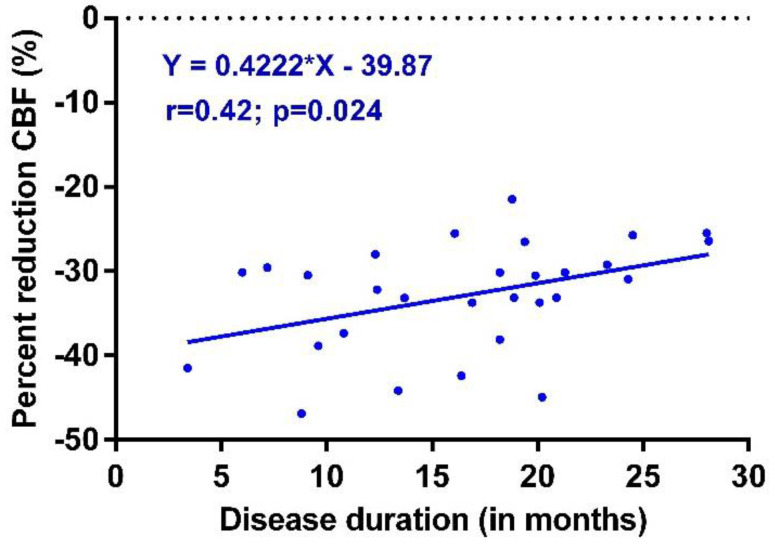
Cerebral blood flow reduction in long-haul COVID-19 patients with POTS, with OH, and with a normal heart rate and blood pressure during the tilt test. CBF: cerebral blood flow; OH: orthostatic hypotension; normHRBP: normal heart rate and blood pressure response during the tilt test.

Figure 3 shows the linear regression of the long-haul COVID-19 disease duration versus cerebral blood flow reduction. The cerebral blood flow reduction diminishes over time after the onset of long-haul COVID-19 symptoms (linear regression: *p* = 0.024).

## 4. Discussion

The main findings of this study are that the incidence of POTS decreases over time after the onset of the long-haul COVID-19 disease, and that an abnormal cerebral blood flow reduction during the tilt test also diminishes over time after the onset of symptoms, but remains abnormal.

POTS has been described in smaller and larger case reports after the onset of the COVID-19 pandemic [1,2,3,4,5,6,7,8,9,10,11,12,13,17,18,19,20]. In our previous study of the first ten long-haul COVID-19 patients, we only observed POTS during a tilt test [13]. In the present study, patients could also develop OH or had a normal heart rate and blood pressure response during the tilt test. This suggests that the POTS reaction may disappear in the time course of the disease and is replaced by another hemodynamic form or may even lead to a normal heart rate and blood pressure response. The normalization of POTS (and also of hypotension) to a normal heart rate and blood pressure during a tilt test have been described previously in three patients [1] and in one patient with POTS, where the heart rate significantly improved after the COVID-19 infection [21]. POTS is a heterogeneous syndrome with variable symptomology between patients, and with multiple mechanisms (hypovolemia, increased inflammatory mediators, excess sympathetic activation, autoantibodies targeting autonomic receptors, small-fiber neuropathy, and tissue laxity in hypermobility) that may produce a similar clinical phenotype, and where there is overlap with other clinical syndromes [22,23]. One can only speculate about the mechanisms that lead to the reduction of the frequency of POTS in the time course after the COVID-19 infection. One of the speculative mechanisms is that the catecholamine production of cytokine-producing immune cells, e.g., the macrophages [24], decreases over time together with the cytokine production. This may lead to a reduction in the frequency of POTS. In addition, the production of autoantibodies, inhibiting vascular receptors, may lead to OH [25]. However, many other mechanisms may be involved and require further investigation.

The second observation described here in this study is that the degree of abnormal cerebral flow reduction is diminished later in time after the onset of the long-haul COVID-19 infection. However, after 25 months, cerebral flow reduction is still grossly abnormal, as evidenced in Figure 3. These values contrast the data of healthy volunteers, where the mean cerebral flow reduction during a tilt test was 7% [16]. This improvement is probably related to the hemodynamic profile differences between the early and late stages of the long-haul COVID-19 disease, mainly POTS early after the onset of COVID-19 versus mainly a normal heart rate and blood pressure response after 24 months (see Figure 2). Long-term follow-up data on the orthostatic intolerance symptoms and cerebral blood flow measurements are not present in this early stage of the pandemic, and studies addressing this question need to be awaited.

The mechanisms underlying the finding that POTS is less frequently observed later in the disease are not clear. Hypothetically, an excess sympathetic drive in the acute phase of COVID-19 may disappear, leading to fewer POTS signs and symptoms [26]. In addition, mechanisms causing chronotropic incompetence may emerge with a longer duration of illness, such as changes in the vagal and sympathetic balance, changes in baroreceptor sensitivity as well as differences in stretching forces, [27,28,29], or changes in myocardial receptor density [30]. This needs to be studied in the future.

It is worthwhile noting that many physicians consider a normal heart rate and blood pressure response to a tilt test as a “normal test”. In the present study of long-haul COVID-19 patients as well as in ME/CFS patients, an abnormal cerebral blood flow reduction was found in patients with a normal heart rate and blood pressure response [16,31]. This has also been observed by others using different cerebral blood flow measurement techniques in other patient groups [32,33,34,35]. The clinical implication is that these patients, without demonstrable tilt test abnormalities with respect to changes in heart rate and/or blood pressure, but with orthostatic intolerance complaints deserve the same attention and therapeutic approaches as patients with demonstrable tilt test abnormalities.

### Limitations

The main limitation is that we inferred from individual patients with variable time intervals after the onset of the COVID-19 disease that the type of hemodynamic abnormalities changes over time. Prospective trials with serial tilt testing of the same patient are needed in a larger group of patients. Furthermore, inclusion bias in the referral, because often patients ask for a referral from their general practitioners after information on social media, may have played a role in the current research. We therefore could not assess the number or percentage of patients with orthostatic intolerance complaints after the infection. We had no or limited information on pre-illness activity levels [36], comorbid disease, or other organ-specific abnormalities in these long-haul COVID patients.

## 5. Conclusions

In long-haul COVID-19 patients with orthostatic intolerance complaints, the hemodynamic abnormalities change over time after the onset of symptoms. Patients studied early after the onset of the disease mainly exhibited POTS during the tilt test, but patients studied later in the time course of the disease showed OH or had a normal heart rate and blood pressure response during the tilt. This suggests that the hemodynamic abnormalities in individual patients may change over time. If confirmed in serial follow-up tilt test studies in these patients, patient management may need to be changed over time. In addition, the objective abnormalities of the orthostatic intolerance, cerebral blood flow measurements, show a reduction in the degree of abnormalities. However, the improvement is modest, and patients still have orthostatic intolerance. Whether this pattern of further improvement is sustained, and results in reduction of orthostatic intolerance complaints, needs to be studied in the future.

## Figures and Tables

**Table 1:** Clinical characteristics of long-haul COVID-19 patients with POTS, with OH, and with a normal heart rate and blood pressure (normHRBP) during the tilt test.

	POTS (*n* = 13)	OH (*n* = 5)	normHRBP (*n* = 11)	*p*-Value
Male/female *	4/9 (31/69%)	1/4 (20/80%)	2/9 (18/82%)	0.75
Age (years)	36 (10)	43 (7)	42 (16)	F (2, 26) = 1.88; *p* = 0.17
Height (cm)	176 (10)	177 (6)	172 (7)	F (2, 26) = 0.86; *p* = 0.44
Weight (kg)	71 (14)	84(11)	71 (17)	F (2, 26) = 1.58; *p* = 0.22
BMI (kg/m^2^)	22.8 (3.6)	26.9 (4.2)	23.9 (5.6)	F (2, 26) = 1.43; *p* = 0.26
BSA (m^2^)	1.86 (0.20)	2.01 (0.11)	1.83 (0.20)	F (2, 26) = 1.54; *p* = 0.23
Disease duration (years) #	16.4 (9.0–18.5)	19.4 (17.5–22.2)	20.9 (12.3–24.5)	X2 (33) = 4.58; *p* = 0.10
OI in daily life yes/no *	13/0 (0/100%)	5/0 (0/100%)	11/0 (0/100%)	1.0

BMI: body mass index: BSA: body surface area (formula duBois); POTS: postural orthostatic tachycardia syndrome; OH: orthostatic hypotension; normHRBP: normal heart rate and blood pressure during the tilt test; OI: orthostatic intolerance; * Chi-square (2 × 2 or 2 × 3 table); # Median with IQR: Mann–Whitney U test.

**Table 2:** Hemodynamic results of long-haul COVID-19 patients with POTS, with OH, and with a normal heart rate and blood pressure (normHRBP) during the tilt test.

	POTS (*n* = 13)	OH (*n* = 5)	normHRBP (*n* = 11)	*p*-Value
HR supine (bpm)	75 (15)	69 (13)	71 (9)	F (2, 26) = 0.47; *p* = 0.63
HR end-tilt (bpm)	110 (21)	95 (14)	86 (12)	F (2, 26) = 6.03; *p* = 0.007; gr 1 vs 3 *p* = 0.006
SBP supine (mmHg)	131 (18)	141 (19)	141 (26)	F (2, 26) = 0.68; *p* = 0.52
SBP end-tilt (mmHg)	126 (22)	109 (15)	139 (28)	F (2, 26) = 2.89; *p* = 0.07
DBP supine (mmHg)	79 (13)	81 (11)	81 (12)	F (2, 26) = 0.07; *p* = 0.93
DBP end-tilt (mmHg)	86 (17)	72 (11)	85 (14)	F (2, 26) = 0.63; *p* = 0.22
PETCO2 supine (mmHg)	40 (3)	37 (2)	39 (2)	F (2, 26) = 2.80; *p* = 0.08
PETCO2 end-tilt (mmHg)	28 (5)	27 (3)	29 (3)	F (2, 26) = 0.88; *p* = 0.43
CBF supine (mL/min)	616 (73)	657 (58)	632 (69)	F (2, 26) = 0.64; *p* = 0.54
CBF end-tilt (mL/min)	396 (70)	459 (39)	441 (61)	F (2, 26) = 2.53; *p* = 0.10

HR: heart rate; SBP: systolic blood pressure; DBD: diastolic blood pressure; PETCO2: end-tidal CO2 pressure; CBF: cerebral blood flow; POTS: postural orthostatic tachycardia syndrome, OH: orthostatic hypotension; normHRBP: normal heart rate and blood pressure during the tilt test.

## Data Availability

Not applicable.

## Appendix A

### Appendix A.1. Tilt Test Protocol to Quantify Orthostatic Intolerance

In short, all participants were examined in a supine position for 20 min before being tilted to 70 degrees [15,16]. The average duration of tilting was 10 min. Heart rate and blood pressure were measured with finger plethysmography. Changes in heart rate and blood pressure during tilt testing were defined previously by Freeman and Sheldon [37,38]. This involved a normal heart rate and blood pressure response, orthostatic hypotension (a decrease of 20 mmHg in systolic blood pressure and more than 30 mmHg in case of systolic blood pressure above 160 mmHg) [39]. A constant increase of at least 30 beats per minute within 10 min of standing, without a significant decrease in blood pressure, was defined as POTS. Syncope involves a temporary loss of consciousness and muscle tone with spontaneous and complete recovery.

### Appendix A.2. Extracranial Doppler Measurements for Determination of Cerebral Blood Flow

The internal carotid and vertebral arteries’ Doppler velocities were obtained by one operator (FCV). Frames were recorded in the supine position for tilting and just before tilting back. The blood flow of the carotid and vertebral arteries was calculated offline by one researcher (CMCvC) who was not familiar with the severity of the disease. Blood flow was calculated for each vessel by multiplying the mean blood flow rate by the blood vessel surface and was expressed in mL/min. Blood flow in the individual arteries was calculated in 3–6 heartbeats and the results were averaged. The total cerebral blood flow was calculated by adding the blood flow of the four arteries together [15,16].
